# Effect of Modified Shaker Exercise on the Amplitude and Duration of Swallowing Sounds: Evidence from Cervical Auscultation

**DOI:** 10.1155/2017/6526214

**Published:** 2017-09-07

**Authors:** Sonia Babu, Radish Kumar Balasubramaniam, Ancy Varghese

**Affiliations:** Department of Audiology & Speech Language Pathology, Kasturba Medical College, Manipal University, Mangalore, Karnataka 575001, India

## Abstract

**Objective:**

Anecdotal evidence shows that the Shaker exercise and its modifications improve pharyngeal muscle contraction. However, there is no experimental evidence for the same. Thus, the present study examined the effect of modified Shaker exercise on the amplitude and duration of pharyngeal muscle contraction using cervical auscultation.

**Design:**

The study follows a cross-sectional study design, where 50 healthy individuals (23 males and 27 females) performed modified Shaker exercise and noneffortful swallow during 10 ml water swallowing. Swallow sound characteristics were analyzed with and without modified Shaker exercise using cervical auscultation.

**Results:**

The results of mixed ANOVA revealed significant differences for the amplitude of swallow sound with modified Shaker exercise (mean = 47.24, SD = 20.64) when compared to noneffortful swallow (mean = 28.19, SD = 10.26) at *p* < 0.05. However, no significant difference was obtained for the swallow sound duration with (mean = 0.19, SD = 0.07) and without (mean = 0.18, SD = 0.07) modified Shaker exercise at *p* > 0.05. No significant difference across the genders was also noted at *p* > 0.05.

**Conclusion:**

The outcomes of the study suggest that modified Shaker exercise improves the amplitude of pharyngeal muscle contraction. Further studies are needed to confirm this finding using gold standard tools like videofluoroscopy.

## 1. Introduction

The Shaker exercise is a series of sustained and repetitive head lifting exercises to enhance the strength of infrahyoid and suprahyoid muscular activity [1, 2]. Shaker exercise includes isometric and isotonic exercises. Isometric exercises are performed by raising the head up for 60 seconds followed by a minute rest for a repetition of three times. Followed by this, isotonic exercises are performed by thirty repetitions of alternate up-and-down movement of the head. This enhances the contraction of the thyrohyoid muscle, strengthens the suprahyoid muscles, facilitates the upward and forward movement of the larynx, and thereby opens the upper esophageal sphincter (UES) [3–5]. Moreover, Shaker exercise also improves both infrahyoid and suprahyoid muscular activity and reduces pyriform sinus residue and backflow aspiration [1, 2]. This exercise has been generally used in cases of oropharyngeal dysphagia due to abnormal UES opening [6].

Shaker et al. have reported a significant increase in the opening of UES with Shaker exercise in dysphagic patients with poor UES opening [7]. Moreover, clinical trials and posttherapy reports from various institutes have reported that the amount of aspiration was significantly less in those clients who performed Shaker exercise than the traditional swallowing therapies [6].

Despite the benefits, Shaker exercise has been found to be physically challenging, especially for elderly individuals with chronic diseases [8]. Recently, several modifications of the Shaker exercise have been proposed such as chin tuck posture against resistance (CTAR), jaw opening against resistance exercise (JOAR), effortful swallow against resistance (ESAR), and working with a swallowing exercise device (e.g., ISO Swallowing Exercise Device, ISO-SED). Research evidence indicated a higher muscular activity with modifications of the Shaker exercise compared with the traditional Shaker exercise [9]. One of the easiest and simplest modifications that this study has attempted is to make the individual sit and apply forehead resistance with the palm. The individual has to extend the head against the resistance applied on the forehead. This modification was attempted in view of upright or standing posture during the normal swallowing process. Moreover, swallowing rehabilitation strategies do suggest upright postures while performing swallows.

Traditional Shaker exercise facilitates physiologic changes in the suprahyoid muscles. Due to its close proximity with the pharyngeal muscles, there is a probability that it augments the pharyngeal muscles and thereby pharyngeal peristalsis. However, the effectiveness of modified Shaker exercise in improving pharyngeal peristalsis is not studied so far. This can be studied objectively by using simple noninvasive procedures such as cervical auscultation. Cervical auscultation is a method of measuring swallow sound using a listening device. The physiologic correlates of the swallowing sounds are thought to reflect the action of pharyngeal walls [10] and considered to be reliable [11–13].

Hence, the present study aimed to investigate the effect of modified Shaker exercise on the amplitude and duration of pharyngeal muscle contraction in healthy young adults using cervical auscultation.

## 2. Materials and Methods

The present study was a cross-sectional study using snowball sampling procedures. 23 males and 27 females in the age range of 18 to 24 years were recruited from the community based on sample size calculations for single group cross-sectional study. All the participants were free from speech, language, and neurological problems affecting their swallowing abilities. None of the participants had any surgical history of the oropharynx. Informed consent was obtained from all the participants included in the study. All the procedures performed in studies involving human participants were in accordance with the ethical standards of the institution.

### 2.1. Procedure

The participants were seated comfortably on a straight back chair in the dysphagia lab of our department and a stethoscope was placed on the lateral side of the neck, above the cricoid cartilage, in front of the sternocleidomastoid muscle and the large vessels [9–11].

**Figure 1:**
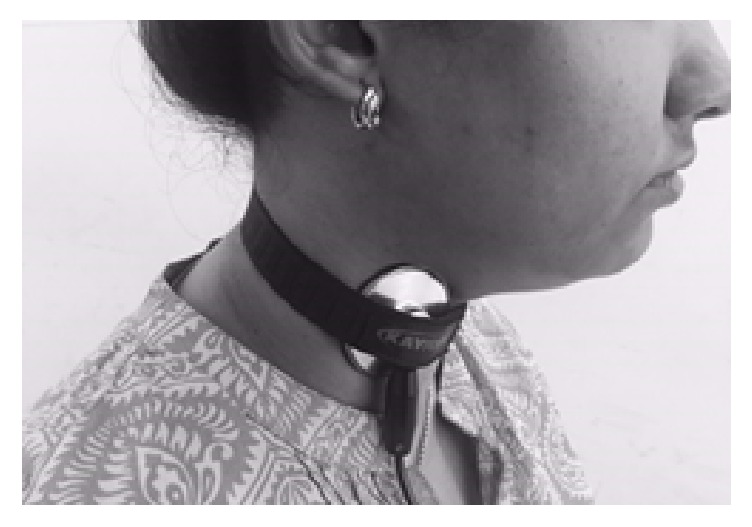
The placement of stethoscope.

Figure 1 shows the placement of the stethoscope for the measurement of amplitude and duration of swallow with and without Shaker exercise.

Examinations were carried out for two types of swallow: swallowing without any effort (noneffortful swallow) and swallowing with modified Shaker exercise. The participants were seated comfortably on a back support chair and 10 ml of water was given for noneffortful swallow in a cup. For the modified Shaker exercise, participants were instructed to extend the head forwards against the applied forehead palm resistance by the therapist and swallow 10 ml of water using a cup in one complete action.

**Figure 2:**
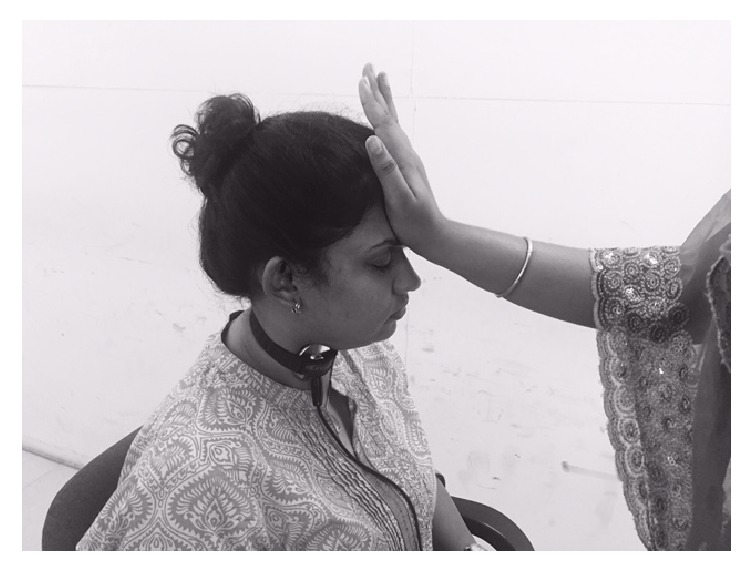
Modified Shaker exercise.

Figure 2 shows the performance of the modified Shaker exercise.

All swallowing examinations were recorded using cervical auscultation module available in the Digital Swallowing WorkStation (Kay Pentax Corporation, Lincoln Park, NJ) connected to the swallowing signal lab. This module captures the swallow sound during swallowing and gives information on amplitude and duration of swallow sounds. Three trials of each task were taken during the procedure. It was ensured that the participants first performed noneffortful swallows followed by modified Shaker exercise. This order was followed to minimize the effect of modified Shaker exercise on the noneffortful swallows.

### 2.2. Data Analysis

**Figure 3:**
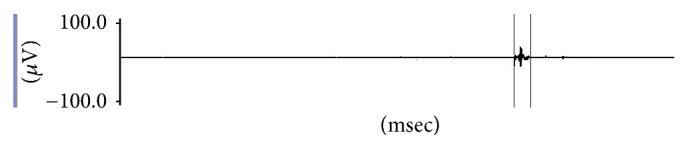
Data analysis of swallow sounds.

Vertical cursors were placed on the onset and offset of the swallow sound for each task as shown in Figure 3 and the outcome measurements such as maximum peak amplitude (*µ*V) and total time duration (msec) were measured. Maximum peak amplitude here refers to the maximum amplitude recorded in the selected interval of swallow sounds waveform whereas the duration of swallow sounds refers to the total time duration measured within the selected interval of swallow sound waveform.

The average of three measurements of each outcome variable was considered for statistical analysis.

### 2.3. Statistical Analysis

The data was then tabulated and subjected to statistical analysis using SPSS (Version 20). Descriptive statistics were used to obtain mean and standard deviation of amplitude and duration of pharyngeal muscle contraction. One-way mixed ANOVA was also carried out, keeping gender as a between-group variable and effect of exercise (with and without exercise) as a within-group variable.

## 3. Results

The present study investigated the effect of modified Shaker exercise on pharyngeal muscle contraction using cervical auscultation. The results of descriptive statistics are shown in Table 1.

The results of one-way mixed ANOVA revealed a significant difference between the means of amplitude for swallow sound with and without modified Shaker exercise (*F*_(1,48)_ = 59.611, *p* < 0.05). However, no significant difference was observed for duration of swallow sounds with and without modified Shaker exercise (*F*_(1,48)_ = 1.886, *p* > 0.05). However, the mean duration of swallow with Shaker exercise showed a slight increase in comparison to without Shaker but was not statistically significant. The results also revealed no significant differences across the genders for amplitude (*F*_(1,48)_ = 0.773, *p* > 0.05) and duration of swallow sounds (*F*_(1,48)_ = 0.370, *p* > 0.05). There was a significant interaction effect for duration of swallow sounds (*F*_(1,48)_ = 4.593, *p* < 0.05). However, no interaction effect was observed for amplitude of swallow sounds (*F*_(1,48)_ = 3.852, *p* > 0.05).

## 4. Discussion

Traditional Shaker exercise has been modified over the years to increase the ease of performance of it in the clinical population. These modifications have been successful in many individuals with dysphagia in the clinical trials. These modifications have significantly decreased aspiration after therapy, reduced the pyriform fossa residue, and so forth. Anecdotal evidence suggests that this exercise would aid in the pharyngeal peristalsis due to the improvement in the suprahyoid muscles. However, this was not experimentally investigated and hence the present study investigated the effect of modified Shaker exercise in healthy young participants using cervical auscultation. Cervical auscultation was chosen as it is noninvasive in nature and the swallow sound characteristics correlate very well with videofluoroscopy [10] and are considered to be reliable [11–13].

In the present study, there was an increase in the mean raw scores of peak amplitude when swallowing with modified Shaker exercise compared with noneffortful swallow. This increase in the peak amplitude could be attributed to the increased elevation of the larynx, thereby facilitating the pharyngeal muscle movement during the modified Shaker exercise. These findings are in consonance with the previous researches supporting suprahyoid muscular activity during the modified Shaker exercise [14]. Research evidence also indicated that the amount of muscular activities is higher in modifications of the Shaker exercise than the traditional Shaker exercise [9, 15].

The results of the duration measures indicated no significant increase in the mean raw scores of duration when swallowing with modified Shaker exercise. Though the muscular activities are higher in the modified Shaker maneuver, these activities did not significantly increase or decrease the duration of swallow sound which may be important for pharyngeal peristalsis. This suggests that modified Shaker exercise did not affect the duration of swallow sounds indicating that duration of pharyngeal muscle contraction is not altered by this exercise.

Results also revealed no significant difference across the genders for amplitude and duration of swallowing sounds. This suggests that there are no gender differences for pharyngeal muscle contraction. However, functional differences in pharyngeal swallowing across the genders have been reported in the literature [16]. Moreover, pharyngeal size is larger in men than in women [17] and, in spite of these differences, pharyngeal muscle contraction as assessed through swallow sound remains the same in the present study.

The results of the present study advocate that modified Shaker exercise can also be used to improve the contraction of the pharyngeal muscles in healthy adults. This exercise improves the amplitude of pharyngeal muscle contraction and would aid in the peristaltic action of pharyngeal muscles. However, it did not affect the duration of pharyngeal muscle activity. Therefore, this exercise would be a promising rehabilitation technique in the management of pharyngeal muscle contraction issues. This information is of great value to the practicing speech pathologists and laryngologists in the management of individuals with reduced UES opening and reduced pharyngeal muscle contraction. However, the present study was carried out in normal individuals and requires experimental investigation in individuals with confirmed pharyngeal paresis/paralysis. Moreover, considering the limitations of cervical auscultation [18], further studies should address similar issues using gold standard techniques like videofluoroscopy. Hence, the results of the present study should be interpreted with caution when applying to a disordered population.

## 5. Conclusion

The present study measured the effect of modified Shaker exercise on pharyngeal muscle contraction in healthy young adults using cervical auscultation. Though it is a preliminary study, results suggest that the modified Shaker exercise is an effective technique in increasing the amplitude of pharyngeal muscle contraction in healthy adults. Further research is warranted to investigate the long-term effect of modified Shaker exercise in healthy adults and individuals with swallowing impairment.

## Figures and Tables

**Table 1:** The mean and SD of amplitude and duration of swallow sound with and without modified Shaker exercise.

Measures	Males (*n* = 23)		Females (*n* = 27)		Total (*n* = 50) (males and females)	
	Mean	SD	Mean	SD	Mean	SD
Amplitude (*µ*V)						
Without Shaker exercise	27.35	11.69	28.91	9.04	28.19	10.26
With Shaker exercise	51.74	21.20	43.42	19.74	47.24	20.64
Duration (msec)						
Without Shaker exercise	0.188	0.08	0.17	0.06	0.18	0.07
With Shaker exercise	0.180	0.08	0.21	0.06	0.19	0.07
